# Suicide among Older Adults with Dementia: Effects of Korea’s Long-Term Care Insurance System

**DOI:** 10.3390/ijerph18126582

**Published:** 2021-06-18

**Authors:** Sungje Moon, Mankyu Choi, Minsung Sohn

**Affiliations:** 1 Research Institute for Healthcare Policy, Korean Medical Association, Seoul 04373, Korea; msjess0329@hotmail.com; 2 BK21 FOUR R&E Center for Learning Health Systems, Department of Health Policy & Management, College of Health Science, Korea University, Seoul 02841, Korea; mkchoi@korea.ac.kr; 3 Department of Health and Care Administration, The Cyber University of Korea, Seoul 03051, Korea

**Keywords:** dementia, suicide, long-term care insurance, older adults, survival analysis

## Abstract

South Korea recently expanded its coverage rate of long-term care insurance (LTCI) by adding a “dementia special grade” in 2014 to improve care service accessibility and extend health life for older adults with dementia. In this study, we propose a multifaceted policy to reduce the suicide risk among older adults with dementia by evaluating the effectiveness of using the long-term care services (LTCS). A sample of 62,282 older adults was selected from the “Older Adults Cohort DB” of the National Health Insurance Service. We conducted Kaplan–Meier and Cox regression to represent the yearly survival curve from 2002 to 2015 according to the individual characteristics. Difference-in-difference estimation was conducted to identify the effect of LTCS on suicide rates by using LTCS before and after 2014. The suicide risk of older adults using LTCS was about 0.256-times lower than those who did not use it (OR = 0.296, 95% CI = 0.183–0.478), whereas it increased after the expansion of the dementia grading (OR = 2.131, 95% CI = 1.061–4.280). To prevent the risk of suicide among older adults with dementia, not only did the mortality rate vary depending on the sex, activities of daily living (ADL), and type of caregiver at the individual level but appropriate national intervention and management, such as improving the accessibility of LTCS, are also needed.

## 1. Introduction

A prolonged lifespan does not necessarily imply an improved quality of life. Although the average life expectancy is increasing worldwide, social and health problems, such as dementia and suicide, have also been on the rise. Considering the rapidly increasing proportion of older adults in the global population, the increasing number of older adults with dementia will soon become a national concern [1]. Dementia is a condition in which an individual’s memory or cognitive functioning deteriorates and progressively interferes with their daily life [2]. 

The number of patients with dementia worldwide is expected to increase from about 50 million today to 152 million in 2050 [3]. In South Korea, the number of patients with dementia was about 710,000 in 2017, 3.6-times higher than in 2007. These indicators suggest that it is necessary to examine not only the medical problems of aging but also the acute public health and sociological problems that older adults face.

Specifically, the risk of suicide increases as dementia progresses, as patients fear the prospect of accelerated physical and mental decline and worry about the emotional and economic burden on their families [4]. In 2016, the global prevalence, incidence, and years lived with disability (YLDs) of dementia were the highest among all mental diseases [5]. Dementia is also associated with depression, anxiety, suicidal thoughts, and self-harm in caregivers, including family and neighbors [6,7]. 

In 2017, the average age-standardized suicide rate in the Organisation for Economic Co-operation and Development (OECD) countries was 11.2 per 100,000 people, with that of South Korea being the highest at 24.6 [8]. Particularly, the leading suicide rate among older adults in South Korea is a problem among OECD countries [9]. The ratio of older adult suicide to the total suicide incidence was over 70% in South Korea, while the average in OECD countries was about 22% [9,10]. Therefore, dementia as a geriatric disease is a pressing concern that requires constant monitoring, prevention, and management from various aspects, as well as continuous research to identify preventive solutions to related social problems, such as suicide.

Recently, a study conducted a systematic review and meta-analysis and confirmed the association of dementia with suicidal ideation, suicide attempt, and completed suicide [11]. According to a follow-up study on suicide among older adults in the long run, older adults with dementia had a three-times higher suicide risk than older adults without dementia [12]. Additionally, the hospitalization rates of older adults with dementia due to self-harm were double those of the rest, and the mortality, re-admission, and hospitalization periods within 12 months after admission were also higher in this group [13]. 

Several studies have verified the cause of suicide in older people with dementia at the individual level [12,13,14,15,16,17,18,19,20,21]. According to the interpersonal theory of suicidal behavior, the most dangerous aspect of suicidal desire is the coexistence of both a thwarted belongingness—feeling disconnected from a social relationship—and a perceived burdensomeness—perceiving oneself as a burden on others [14]. Patients with dementia feel alienated from social events and conversations concerning family or friends, and think that they are not helpful or are burdensome to others due to their memory loss [15]. In other words, such patients are a vulnerable group exposed to suicidal thoughts and risks due to worries about the characteristics of the disease. 

Among the older adults who committed suicide due to dementia, the overall suicide attempts and suicide rates were higher among patients with mild dementia who had been recently diagnosed [14,16,17,18]. Similarly, suicide attempts among these patients were higher than among older adults with light cognitive impairment [19,20,21]. Studies on suicide among older adults with dementia have mainly considered socioeconomic factors at the individual level and are limited in presenting specific guidelines for prevention at the national level. Since dementia is difficult to cure and rarely achieves full recovery, unlike other diseases, institutional help, such as early detection and management of disease, is essential to lower the suicide rate.

According to the World Health Organization (WHO), suicide is a representative preventable death, requiring appropriate national intervention and management. Suicide can also be prevented through effective health policy and medical services [22]. As a strategy to respond to suicide risk factors, steps should be taken to improve the medical accessibility and mental health for all citizens, along with selective prevention and follow-up measures for suicide-vulnerable groups that do not receive adequate community support. In a report titled the “Development of National Strategies for Suicide Prevention”, the United Nations and WHO recommended that the government pay attention to suicidal behavior, and emphasized comprehensive policies, including social solidarity and responsibility. Dementia, which is a high-risk factor for suicide, causes suffering in both patients and families, and therefore the government needs to alleviate the pain and burden of the patients and their families by expanding the dementia management policy and the social safety net.

In Korea, the long-term care insurance (LTCI) system was introduced in 2008, and since then the number of users of the service has grown, increasing by 15.1% to about 770,000 older adults who received grading as of 2019 [23]. In particular, to improve accessibility for older people with dementia, a “dementia special grade” was established in the LTCI system in 2014. Additionally, the expansion of day and night shelters for dementia patients improved accessibility, relieved the burden of care for patients with mild dementia, and improved their quality of life. 

Further, South Korea announced a “National Responsibility Plan for Dementia” to reduce the out-of-pocket costs for patients with severe dementia by 10%. These policies aim to protect patient families from the economic and care-giving burdens of dementia and social problems, such as suicide. In a recent study, a cost-effectiveness assessment concluded that the long-term care insurance system reduced the lifetime medical expenses of older people with dementia [24].

Previous studies have researched the association between policies at the national or community level, such as long-term care services (LTCS) and suicide rates in older adults with dementia. A study on suicide prevention programs, conducted for older adults with mild dementia in South Korea who received day care services, reported a positive effect on their subjective health level, depression, and suicidal intentions [25]. Additionally, admission to a nursing home was found to lower the suicide risk among those with dementia aged above 60 years [4]. 

According to a study conducted on 634 older adults with dementia for seven years, about 10% of them had thought of suicide [26]. Prior research has mainly focused on the previous processes of suicide, such as depression and suicidal intents, rather than the final outcome of suicide in older people with dementia. Further, some studies mainly focused on the caregivers of dementia patients, such as their tendency to develop depression, stress, or murderous behaviors or impulses [6,27]. Therefore, in this study, we emphasize national-level policies to protect older adults with dementia and suggest a direction for suicide-prevention strategies.

For suicide prevention, it might be necessary to approach vulnerable targets that are more exposed to the risk and environmental factors of suicide. Additionally, we need to devise measures that can prevent suicide by supplying appropriate and timely help and institutional assistance. Therefore, we evaluate whether a national approach through the LTCS can prevent suicide among older adults with dementia, and we propose a multifaceted policy to prevent suicide. First, the risk factors of suicide among Korean older adults with dementia were identified at the individual level. Second, the effect of using LTCS on suicide among older adults with dementia was determined at the national level. Lastly, the effectiveness of policies, such as the introduction of LTCI in 2008 and the expansion of the dementia grade in 2014, was evaluated.

## 2. Materials and Methods

### 2.1. Research Design and Participants

In 2002, the National Health Insurance Service (NHIS) in South Korea established the ‘National Health Information Database (NHI DB)’, which includes information on patient medical records, disease history, prescriptions for health insurance, and medical aid beneficiaries. The DB was systematically sampled and stratified by sex, age, region, and the level of insurance cost to ensure representativeness. Additionally, the NHI DB is divided into ‘Standard Cohort’, ‘Health Examination Cohort’, ‘Older Adults Cohort’, and ‘Infant Cohort’ DBs to study specific populations.

This study used the ‘Older Adults Cohort DB’ from 2002 to 2015. This DB randomly extracted 558,147 randomly extracted people, who constituted about 10% of the total 5.5 million Korean older adults aged over 60 in December 2002. Therefore, the cohort DB was tracked since 2002 and followed up to 2015 in 2020. To obtain a study sample of older adults with dementia, we employed the following four steps. 

**Figure 1:**
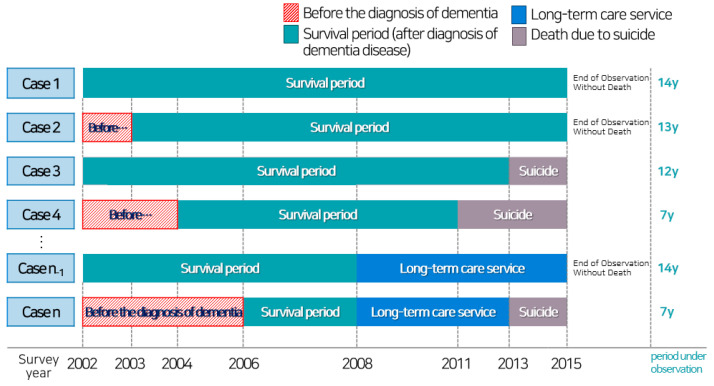
The structure of the study sample.

Step 1: We selected 121,235 older adults who were outpatients or inpatients at least once with a disease code related to dementia (F00–F03, the Korean standard classification of disease and cause of death, KCD) from 2002 to 2015 (in Figure 1, the “survival period (green box)” refers to the duration of the dementia experience). Step 2: All other causes of death, except suicide (which is a dependent variable in this study), were deleted. Step 3: To meet the baseline of health conditions of older adults in each group according to the type of LTCS, older adults with LTCS grading were selected based on their score for long-term care approval; those who had been judged to need LTCS after the introduction of the LTCI system in 2008 were selected. Step 4: The final sample included 62,282 older adults after creating a balanced panel for 14 years and excluding individuals with missing values for the main variables used in the analysis.

We visualized how the samples selected for this study were structured in Figure 1. First, among the older adults who survived until the end of follow-up, Case 1 refers to older adults who had been diagnosed with dementia from the beginning of the observation, while Case 2 comprises older adults diagnosed with dementia during the observation (i.e., Case 1 was diagnosed with dementia in 2002 and survived until 2015; Case 2 was diagnosed with dementia between 2003 and 2008 and survived until 2015). Second, Cases 3 and 4 had the same criteria for period of dementia diagnosis as Cases 1 and 2; however, the difference was that, in the latter cases, the older adults died before the end of follow-up (i.e., Case 3 was diagnosed with dementia in 2002 and committed suicide before 2015; Case 4 was diagnosed with dementia between 2003 and 2008 and committed suicide before 2015). 

Lastly, Case n_-1_ and Case n were older adults who had the same criteria as Cases 1–4 but represent those who used services under the LTCI since its introduction (i.e., Case n_-1_ was diagnosed with dementia in 2002 and used LTCS after 2008. Different cases depend on whether the case committed suicide or not; Case n was diagnosed with dementia between 2003 and 2008 and used LTCS after 2008. Different cases depend on whether the case committed suicide or not.).

### 2.2. Measures

#### 2.2.1. Dependent Variable: Suicide

Suicide was the dependent variable in this study, to verify whether the cause of death data included the suicide codes (X60–X84). The binary variable was coded 1 if the older adult had committed suicide and 0 otherwise.

#### 2.2.2. Independent Variable: Long-Term Care Services

This study took the experience of using LTCS as a policy variable to confirm the effect of LTCS on preventing suicide among the older adults with dementia. The LTCS types were classified into non-user, facility benefits, and in-home benefits. Generally, facility services are used when the disease symptoms are severe. The LTCI provides three types of services (LTCS): facilities, in-home, and cash services. In severe cases or grades of 1 to 3, facility services can be used; by contrast, for grades 4 to 5 and the dementia special grade, patients mainly use in-home services. Therefore, the service type was classified as a facility service if the older adult used both facilities and in-home services. We excluded cash services because the frequency of using cash services is very small.

#### 2.2.3. Control Variables: Demographic Characteristics and Health Status

The control variables in this study included the demographic characteristics of older adults with dementia, that is, sex, age, and income, as well as caregivers and their health status. Sex comprised male and female, and their ages were classified into three categories of 60–69 years, 70–80 years, and over 80 years. The NHI premium for the tenth quantile was used as a proxy for income level because NHI coverage in Korea is subject to compulsory payment of health insurance premiums based on individual income levels. 

This variable was re-categorized into five quantiles: first quantile (medical aid beneficiaries), second quantile (the first–second quantile, which is the lowest income level), third quintile (third–fifth), fourth quintile (sixth–eighth), and fifth quintile (ninth–tenth). The types of caregivers were categorized into family and neighbors, professional caregivers, and no caregivers. Regardless of what LTCS older people used, they may choose professional caregivers or family caregivers, or they may not use caregivers. The health status included activities of daily living (ADL) and cognitive ability. 

The ADL was classified into three categories (independent, partially dependent, and fully dependent) by summing the scores for 13 questions (activities): bathing, washing the face, washing hair, brushing teeth, eating, dressing, transfer, sitting up, moving to side sit, going out of the room, going to the toilet, controlling urine, and controlling feces, on the basis of four responses (full independence, incomplete independence, partial dependence, and full dependence). For cognitive ability, the binary categories were divided into “good” and “bad” using the total scores of 10 questions: impairment of long-term or short-term memory, communication, counting, awareness of routines, decline in judgment, inability to recognize instructions, date, place, and age.

### 2.3. Analysis Strategy

The following analysis methods were used. First, we conducted a chi-square test to identify the factors affecting suicide among older adults with dementia. Second, the Kaplan–Meier and Cox regression analyses were conducted to create the yearly survival curve from 2002 to 2015, and the suicide rate was identified by demographic characteristics, health status, and policy. Third, to confirm the effect of reinforcing insurance coverage with the expansion of LTCS’s “dementia special grade”, a difference-in-differences (DID) estimation was conducted to analyze the effect of LTCS on suicide rates by comparing statuses before and after 2014. 

Model 1 analyzed the effect of the interaction term (before and after expansion x service was used or not) on the suicide rate, and model 2 confirmed the effect of the interaction term on the suicide rate after adjusting the control variables. This study used a quasi-experimental design to evaluate the impact of the expansion of LTCI’s “dementia special grade”. In addition to DID analysis, we analyzed panel data from 2009 to 2015 after the introduction of the LTCI system. The effect was estimated by calculating the pre (2009 to 2013)–post (2014 to 2015) difference in outcomes for the policy group (LTCS users) and the pre–post difference in outcomes for the reference group (non-LTCS users).

## 3. Results

### 3.1. Differences in Suicide Frequency by General Characteristics among the Older Adults with Dementia

Table 1 shows the results of the chi-square test to compare the difference in suicidal death rates according to the general characteristics of older adults with dementia. The annual average suicidal mortality rate in the study sample was 25.94 older adults per 100,000 populations. The frequency of suicide by males (0.33%) was more than twice that of females (0.14%), and by age, the suicide rate was highest in those in their 60s (0.41%), followed by those in their 70s (0.21%), and those who were above 80 years old (0.15%). Concerning ADL, suicide frequency was the highest in those who were independent (0.3%) and was the lowest in fully dependent older adults (0.05%). 

Additionally, older adults with good cognitive ability (0.27%) had a higher suicide frequency than those with poor cognitive ability (0.11%). Considering the type of caregiver, older adults with no caregiver had the highest suicide frequency (0.35%), followed by those with family or neighbors as caregivers (0.23%), and those with nurses as caregivers (0.1%). Lastly, LTCS non-users (0.33%) had a higher suicide frequency than users, and in-home service users (0.23%) had a higher suicide frequency than users of facility services (0.06%). The difference in suicide frequency according to income level was not statistically significant.

### 3.2. Factors Affecting Suicide among Older Adults with Dementia

Using the Cox proportional hazard model, this study confirmed the hazard ratio of suicide according to the type of LTCS used by older adults with dementia. Table 2 shows the analysis results. 

Model 1 is the result of analyzing the suicide hazard ratio according to the type of LTCS without adjusting the control variables. Compared to the older adults who did not use LTCS, the suicide rate among those using facility services was about 0.628-times lower (*p* = 0.017), while the suicide rate among those using in-home services was about 0.151-times lower (*p* < 0.001).

Model 2 is the result of adjusting controls for the suicide hazard ratio according to LTCS types. The hazard ratio of each control variable on suicide was also verified. As a result of adjusting demographic characteristics and health status, the suicide risk of older adults with dementia who used facility services was about 0.256-times lower than those who did not use LTCS (*p* < 0.001). However, the effect of in-home service on suicide risk was not statistically significant. Among the control variables, the hazard ratio according to sex and ADL was significant. The suicide hazard ratio of women was 0.397-times lower than that of men (*p* < 0.001), and the hazard ratio of fully dependent older adults with dementia was 0.29-times lower than that of independent older adults (*p* < 0.001).

The results of the Kaplan–Meier Survival Curve, which estimates the survival rate during the 14 years, are presented in Appendix A to show the univariate effect of all variables (i.e., demographic characteristics, health status, and use of LTCS) used in the Cox proportional hazard regression on mortality. Statistically significant differences were found based on sex, ADL, cognitive ability, caregivers, and use of LTCS (log-rank test *p* < 0.001). By contrast, there was no difference in the survival rate by suicide according to age (*p* = 0.673) and income level (*p* = 0.883).

### 3.3. Policy Effects of Long-Term Care Services on Suicide among Older Adults with Dementia

Table 3 shows the results of the DID analysis performed to compare the policy effects of the LTCS’s coverage expansion of the “dementia special grade” on the suicide rate of older adults with dementia. The suicide rate of older adults with dementia increased in the period after the expansion of dementia grade compared to that before the change (OR = 2.131, 95% CI = 1.061–4.280, *p* = 0.034). However, it was lower among LTCS users compared with among non-users (OR = 0.296, 95% CI = 0.183–0.478, *p* < 0.001). The interaction term indicating whether older adults with dementia used LTCS after the expansion of the dementia grading was not statistically significant.

## 4. Discussion

In this study, we verified the risk factors of suicide among older adults with dementia at the individual level and evaluated whether the national intervention of LTCS could prevent suicide among older adults with dementia in the context of a continuously aging population and the rapidly increasing mortality rate of older adults with dementia. Despite Korea’s high and growing suicide rate among people with dementia, there is a lack of empirical research on suicide. This study analyzed the data of 62,282 older adults with dementia from the total population of individuals aged over 60 years in Korea through random sampling, using data from the “Older Adults cohort DB” from 2002 to 2015.

First, at the individual level, the results of the analysis of risk factors affecting suicide in older adults with dementia were as follows: men had a higher suicide rate than women, and the suicide rate among older adults with dementia was higher when their ADL and cognitive functioning were mild rather than severe. Additionally, the suicide rate was the highest when there were no caregivers to help them with daily living. The suicide rate was also higher if family members or acquaintances cared for them instead of professional caregivers.

Regarding ADL and cognitive functioning, the suicide rate among older adults with dementia was higher when their ADL and cognitive functioning were mild rather than severe. This is consistent with recent studies showing that older adults diagnosed with dementia very recently are more likely to attempt suicide [4,14,16,17,18,19,20,21]. In Korea, a study assessing the effects of “a suicide prevention program” for older adults with mild dementia showed positive effects on perceived health conditions, social support, depression, and suicidal intentions over time [25]. 

Therefore, it is essential to provide interventions to prevent the risk of suicide among older adults with mild dementia and to provide health education and rehabilitation programs for mental health management [28]. Additionally, a non-pharmacological strategy, such as multicomponent training, may be an important means to enhance ADL functions in older adults with dementia [29]. Recently, the Korean government has also been making efforts to invest in various programs in the LTCS facility and offer customized services according to the severity of diseases.

Regarding the caregivers of older adults with dementia, the rate of suicide risk was lower when professional caregivers were used rather than family members or acquaintances. From the literature, we can infer that the reason for this is that patients’ perception of being a burden and stress increase when family members or close neighbors are responsible for caregiving. In Korea, there are several cases of older adults suffering from dementia committing suicide in the early stages because of their inability to overcome the mental distress of being a burden on the family [30]. In addition, care by caregivers can also have a positive effect on the formation of relationships between the patient and the caregiver. 

Thus, the satisfaction of caregivers’ self-care needs and behaviors, including ample sleep, social engagement and support, and leisure activities, are also essential in the health care of patients with dementia [31,32]. Systematic support for the formation of a relationship between dementia patients and family caregivers can have a positive effect on both the patients and family caregivers [33]. A Canadian study reported that a positive social relationship alone could reduce intentional self-harm among long-stay home care clients aged over 60 years [34]. Additionally, one reason for the lower risk of suicide when using professional caregivers rather than family members and acquaintances, relates to problems with the caregiver’s expertise as well as the burden of support for family or acquaintances. 

Therefore, it is essential to develop a “specialized curriculum” focused on staff delivery standards and to ensure mandatory training for unskilled nurses, as in Japan, to provide standardized services and establish the infrastructure for ascertaining the role of caregivers [35,36]. Moreover, older adults with dementia and their caregivers can expect targeted improvements, such as clinical knowledge of the disease, better communication between dementia patients and service providers, and more proactive care plans through systematic counseling and education for an acknowledged cognitive decline [37].

Second, an examination of the effects of LTCS intervention at the national level revealed that, among older adults with LTCI grading who needed LTCS, the suicide rate of non-users was higher than that of LTCS users. Among users, by the type of LTCS, the suicide rate of home-service users was higher than that of facility service users. In other words, LTCS provided by the government had a positive effect on the prevention of suicide among older adults with dementia. 

This is consistent with the results of previous studies in that the effects of a national system or community program could contribute to the mental health of dementia patients [38,39]. Older people also experience depression as they age and feel isolated, especially when they perceive themselves as being burdensome to others [14,15]. Thus, they have a lower suicide rate when they are protected in a facility and cared for in a space where communication with the people around them is relatively high.

Additionally, DID analysis of the effects of the LTCI system on older adults with dementia showed that the suicide rate among users of the system was significantly lower than that of non-users, depending on the services used. In other words, the use of LTCS had a positive effect on lowering the suicide rate among older adults with dementia. Therefore, the country’s policy direction to increase access to LTCS might be the most appropriate direction. 

Furthermore, efforts are required to establish various programs and a care service culture within the LTCI system, as well as a quantitative increase in nursing facilities, to prevent suicide among older adults with dementia. Particularly, as in-home service users had the highest suicide rate, it is necessary to develop measures to ensure continuous management and protection by recognizing problems with long-term home care and establishing in-home monitoring systems by utilizing the various apps and chatbots that have recently emerged.

The suicide rate was higher after the expansion of the LTCS grading (after 2014) compared to that before the expansion (before 2014). Thus, there were no significant results regarding the relation between suicide rates before and after the expansion of the grading and whether the LTCS was used. The LTCI system has increased the accessibility of services for older adults with dementia by introducing a special dementia grade since 2014; however, no change in the suicide rate was observed in this study. 

This may be caused by an increase in the inflow of older patients with dementia at a high risk of suicide who entered the system in the early stages of enhanced system security. In fact, in order to observe policy effects, in many policy studies, analysis is not conducted at the time when the scheme is introduced, to exclude the effect of amplified change due to the introduction [40]. There may be limitations due to the shorter observation duration of the system; therefore, continuous monitoring and research are needed to develop measures to prevent and manage patients with dementia in the future.

## 5. Conclusions

This study is meaningful as it is the first empirical study to verify the effectiveness of LTCS on suicide among older adults with dementia in South Korea. This study found that at the individual level, suicide rates were more statistically significant among men, older adults with mild dementia symptoms who had no caregivers or had family/neighbor caregivers. We also confirmed that the use of LTCS could reduce the suicide rate of older adults with dementia. Thus, the use of LTCS strengthened the accessibility of the service for older adults with dementia and contributed to the prevention of suicide by providing care services, indicating the future policy direction.

However, the effect of enhancing the coverage by expanding the “dementia special grade” of LTCI system was limited to a short-term observation over only two years because the comparison was based on the situation in 2014 when the policy was expanded. Therefore, future research should undertake a continuous assessment of the LTCI system from a long-term perspective. This study verified that LTCS had a preventive effect on suicide in older people with dementia, and, for more effective suicide management, developing suicide prevention programs within the LTCI system should be considered in the future.

## Figures and Tables

**Table 1:** Suicide frequency by general characteristics of the elderly with dementia.

Suicide		Yes ^1^		No		Chi-Square
		N	%	N	%	
Sex	Male	56	0.33	17,083	99.67	22.82 ***
	Female	63	0.14	45,080	99.86	
Age	60–69	14	0.41	3442	99.59	11.16 **
	70–79	55	0.21	26,220	99.79	
	Over 80	50	0.15	32,501	99.85	
Income ^2^	Fifth quintile (Highest)	37	0.18	21,096	99.82	0.68
	Fourth quintile	25	0.19	12,860	99.81	
	Third quintile	11	0.18	6114	99.82	
	Second quintile	24	0.21	11,278	99.79	
	First quintile (Lowest)	22	0.20	10,815	99.80	
ADL ^3^	Independent	75	0.30	24,975	99.70	38.27 ***
	Partially dependent	33	0.21	15,471	99.79	
	Fully dependent	11	0.05	21,717	99.95	
Cognitive ability ^4^	Good	86	0.27	31,371	99.73	22.59 ***
	Bad	33	0.11	30,792	99.89	
Caregivers	None	14	0.35	4001	99.65	17.99 ***
	Family/Neighbor	82	0.23	35,367	99.77	
	Nurse	23	0.10	22,795	99.90	
LTCS ^5^	Non-user	55	0.33	16,603	99.67	39.99 ***
	Facility	14	0.06	23,630	99.94	
	In-home	50	0.23	21,930	99.77	
Total		119	0.19	62,163	99.81	

^1^ The annual average of suicidal mortality rate in the study data was calculated as 25.94 elderly per 100,000 populations. Korea’s dementia mortality rate in 2015 was 18.6 per 100,000 population (Statistics Office); the study sample is overestimated because it is likely to be a health risk group as LTCS applicants are among the elderly with dementia. ^2^ Income level: first quintile (Medical aid beneficiaries), second quintile (The NHI premium for the tenth quantile: first–third), third quintile (fourth–fifth), fourth quintile (sixth–eighth), and fifth quintile (ninth–tenth). ^3^ Activity Daily Limitation: Independent (score 13–19), partially dependent (score 20–26), and fully dependent (score 27–39). ^4^ Cognitive ability: Good (score 0–5) and Bad (score 6–10). ^5^ LTCS: Long-Term Care Service. ** *p* < 0.01, *** *p*-value < 0.001.

**Table 2:** Cox proportional hazard regression for LTCS.

		Model 1			Model 2		
		HR	95% CI	*p*-Value	HR	95% CI	*p*-Value
LTCS ^1^	Non-user	1			1		
	Facility	0.151	0.084–0.271	<0.001	0.256	0.136–0.482	<0.001
	In-home	0.628	0.428–0.921	0.017	0.732	0.487–1.100	0.133
Sex	Male				1		
	Female				0.397	0.275–0.574	<0.001
Age	60–69				1		
	70–79				0.833	0.458–1.516	0.550
	Over 80				0.991	0.533–1.843	0.978
Income ^2^	Fifth quintile (Highest)				1		
	Fourth quintile				1.146	0.689–1.905	0.600
	Third quintile				1.147	0.584–2.250	0.691
	Second quintile				1.447	0.864–2.426	0.161
	First quintile (Lowest)				1.167	0.675–2.015	0.581
ADL ^3^	Independent				1		
	Partially dependent				0.981	0.630–1.528	0.933
	Fully dependent				0.290	0.145–0.579	<0.001
Cognitive ability ^4^	Good				1		
	Bad				0.737	0.474–1.145	0.174
Caregivers	None				1		
	Family/Neighbor				0.634	0.348–1.158	0.138
	Nurse				0.554	0.276–1.111	0.096

^1^ LTCS: Long-Term Care Service. ^2^ Income level: first quintile (Medical aid beneficiaries), second quintile (The NHI premium for the tenth quantile: first–third), third quintile (fourth–fifth), fourth quintile (sixth–eighth), and fifth quintile (ninth–tenth). ^3^ Activity Daily Limitation: Independent (score 13–19), partially dependent (score 20–26), and fully dependent (score 27–39). ^4^ Cognitive ability: Good (score 0–5) and Bad (score 6–10). ※ facility (reference): non-user (HR = 3.910, 95% CI = 2.073–7.373) and in-home (HR = 2.862. 95% CI = 1.512–5.417).

**Table 3:** DID analysis of expansion of LTCS with dementia special rates.

		OR	95% CI	*p*-Value
Expansion of dementia special rates	Before (–2013)	1		
	After (2014–)	2.131	1.061–4.280	0.034
LTCS ^1^	Non-user	1		
	User	0.296	0.183–0.478	<0.001
Interaction term	After expansion × Use	1.589	0.667–3.788	0.296
Sex	Male	1		
	Female	0.418	0.290–0.603	<0.001
Age	60–69	1		
	70–79	0.488	0.271–0.880	0.017
	Over 80	0.430	0.237–0.781	0.006
Income ^2^	Fifth quintile (Highest)	1		
	Fourth quintile	1.122	0.675–1.866	0.657
	Third quintile	1.041	0.530–2.042	0.908
	Second quintile	1.283	0.766–2.150	0.343
	First quintile (Lowest)	1.095	0.635–1.889	0.744
ADL ^3^	Independent	1		
	Partially dependent	1.252	0.785–1.999	0.346
	Fully dependent	0.380	0.187–0.774	0.008
Cognitive ability ^4^	Good	1		
	Bad	0.824	0.527–1.288	0.396
Caregivers	None	1		
	Family/Neighbor	0.812	0.451–1.462	0.488
	Nurse	0.604	0.301–1.212	0.156

^1^ LTCS: Long-Term Care Service. ^2^ Income level: first quintile (Medical aid beneficiaries), second quintile (The NHI premium for the tenth quantile: first–third), third quintile (fourth–fifth), fourth quintile (sixth–eighth), and fifth quintile (ninth–tenth). ^3^ Activity Daily Limitation: Independent (score 13–19), partially dependent (score 20–26), and fully dependent (score 27–39). ^4^ Cognitive ability: Good (score 0–5) and Bad (score 6–10).

## Data Availability

The data that support the findings of this study are available from ‘Older Adults Cohort DB (2002 to 2015)’ for the longitudinal study of aging by the National Health Insurance Service at https://nhiss.nhis.or.kr/bd/ay/bdaya001iv.do (accessed on 15 May 2020). Restrictions apply to the availability of this data, which was used under license for the current study, and so is not publicly available. However, the data can be available for a fee if the author receives permission from the National Health Insurance Service following the IRB approval.

## Appendix A

Suicide survival curves by influencing factors (a. sex, b. age, c. income, d. ADL, e. cognitive ability, f. caregivers, g. LTCS) of the older adults with dementia.
